# Feasibility of point-of-care cardiac ultrasound performed by clinicians at health centers in Tanzania

**DOI:** 10.1186/s12872-021-02045-y

**Published:** 2021-05-12

**Authors:** Delilah Kimambo, Samuel Kennedy, Engerasiya Kifai, Neema Kailembo, Christie Eichberg, Sarah Markosky, Ishan Shah, Eric Powers, Peter Zwerner, Susan E. Dorman, Mohamed Janabi, Richard Bayer

**Affiliations:** 1 Jakaya Kikwete Cardiac Institute, Dar es Salaam, Tanzania; 2 Medical University of South Carolina, 135 Rutledge Avenue, Room 1207, Charleston, SC 29425 USA

**Keywords:** Echocardiography, Transthoracic, Point of care technology, Training

## Abstract

**Background:**

Point-of-care cardiac ultrasound (cardiac POCUS) has potential to become a useful tool for improving cardiovascular care in Tanzania. We conducted a pilot program to train clinicians at peripheral health centers to obtain and interpret focused cardiac POCUS examinations using a hand-held portable device.

**Methods:**

Over a 5-day period, didactic and experiential methods were used to train clinicians to conduct a pre-specified scanning protocol and recognize key pathologies. Pre- and post-training knowledge and post-training image acquisition competency were assessed. In their usual clinical practices, trainees then scanned patients with cardiovascular signs/symptoms, recorded a pre-specified set of images for each scan, and documented their interpretation as to presence or absence of key pathologies on a case report form. A cardiologist subsequently reviewed all images, graded them for image quality, and then documented their interpretation of key pathologies in a blinded fashion; the cardiologist interpretation was considered the gold standard.

**Results:**

8 trainees (6 Clinical Officers, 1 Assistant Medical Officer, and 1 Medical Doctor) initiated and completed the training. Trainees subsequently performed a total of 429 cardiac POCUS examinations in their clinical practices over a 9 week period. Stratified by trainee, the median percent of images that were of sufficient quality to be interpretable was 76.7% (range 18.0–94.2%). For five of eight trainees, 75% or more of images were interpretable. For detection of pre-specified key pathologies, kappa statistics for agreement between trainee and cardiologist ranged from − 0.03 (no agreement) for detection of pericardial effusion to 0.42 (moderate agreement) for detection of tricuspid valve regurgitation. Mean kappa values across the key pathologies varied by trainee from 0 (no agreement) to 0.32 (fair agreement).

**Conclusions:**

The 5-day training program was sufficient to train most clinicians to obtain basic cardiac images but not to accurately interpret them. Proficiency in image interpretation may require a more intensive training program.

**Supplementary Information:**

The online version contains supplementary material available at 10.1186/s12872-021-02045-y.

## Introduction

Cardiovascular diseases (CVD), incuding coronary artery and structural/valvular heart disease, are a major cause of morbidity and mortality in sub-Saharan Africa. In 2013 an estimated one million deaths in sub-Saharan Africa were attributable to CVD, and there was an almost two-fold increase in the overall number of CVD-related deaths during the period of 1990–2013 [1]. In 2018, sub-Saharan Africa represented the only geographical region in world where the rate of death from CVD was increasing [2]. Similarly, the World Health Organization (WHO) has previously estimated the prevalence of rheumatic heart disease to be 5.7 cases per 1000 children in Sub-Saharan Africa, although subsequent ultrasound-based imaging studies suggest a much higher rate of disease between 15 and 30 cases per 1000 [3, 4]. The increasing burden of CVD is commensurate with changing social and socioeconomic factors as well as shifts in population dynamics [5–7]. Accompanying this need for improved detection of CVD is the need for strengthening the referral system so that patients are triaged in a manner that optimizes population health. In Tanzania, the Jakaya Kikwete Cardiac Institute (JKCI) located in Dar es Salaam serves as the national referral center and has the capacity for advanced cardiac care, but there are needs for strengthening the screening, identification, and management of CVD at other levels of the health service pyramid.

By facilitating early disease recognition, point-of-care ultrasound (POCUS) has potential to become a useful tool for improving health in middle tiers of the health care system in Tanzania. Recent studies conducted in resource-limited settings have shown that non-physician clinicians can be trained to proficiently perform focused antenatal ultrasounds and to accurately identify key abnormal findings [8–11]. This begs the question as to whether a similar approach can be used for focused cardiac POCUS. In the U.S. and other developed healthcare systems, cardiac POCUS using portable devices has been shown to provide clinically meaningful information compared to physical examination alone, and POCUS is rapidly expanding in use in cardiology, emergency, and critical care medicine [12–15]. A small body of research supports the feasibility of training medical students and non-specialist physicians to acquire and interpret cardiac ultrasound images, but most of these studies have been performed in well-resourced settings [16–20]. Additionally, while there has been evidence demonstrating the utility of cardiac POCUS in more resource limited settings; these studies focused on the training of physicians [21, 22]. We recently surveyed clinicians in the Dar es Salaam environs with regard to training priorities, and cardiac POCUS was consistently identified as an area of interest and need.

Given these needs, opportunities, and clinicians’ interest in POCUS, we conducted a pilot program to train clinicians, predominately Clinical Officers, working in health centers to perform and interpret focused cardiac ultrasound examinations. The content and outcomes of training are described here.

## Methods

### Selection of trainees

This study was conducted in Kisarawe district on the eastern coast of Tanzania. The District Medical Officer selected interested general clinicians who were at the professional level of Clinical Officer or above at health centers within the district. Within the Tanzanian health system Clinical Officers have undergone a 3-year (2 years of course work with 1 year of clinical clerkships) training program that leads to a Diploma in Clinical Medicine. Within the health system in Tanzania, the Clinical Officer scope of practice includes family and emergency medicine without immediate supervision in the most peripheral sectors of the health system, namely dispensaries and health centers. Physicians are regularly available at the district hospital, but cardiologists are regularly available only in higher level hospitals in large cities.

### Training process and content

An adult learning approach was used and included didactic and practical sessions. All trainees attended the same in-person training over a 5-day period, Monday through Friday approximately 9 a.m. to 5 p.m. daily. Didactic sessions including PowerPoint presentations comprised approximately 20% (7 h total) of the training period and focused on basic cardiac anatomy and physiology, introduction to cardiac ultrasound, the focused cardiac scanning protocol, the normal cardiac ultrasound, and identification of key cardiac abnormalities using ultrasound. Experiential hands-on learning sessions comprised approximately 70% (24 h) of the training period and were supervised by the three trainers (a registered cardiac sonographer with 9.5 years of experience [SM] and two cardiologists specializing in echocardiography [RB and DK]). Initially the trainees performed supervised scans of six healthy adults known to have normal cardiac ultrasound examinations, in two 3-h sessions. Subsequently, they performed supervised scans of eight assenting adults who were drawn from the inpatient wards and outpatient clinics at JKCI and known to have abnormal cardiac ultrasound examinations. Six patients had abnormal LV systolic function, four had some degree of mitral regurgitation, two had aortic insufficiency, six had some degree of tricuspid regurgitation, and 2 had some degree of pericardial effusion.

The ultrasound scanning protocol, in sequence, included four views with seven images: parasternal long axis 2D with and without color-flow Doppler over the mitral and aortic valve simultaneously, apical 4 chamber 2D with and without color-flow Doppler over the mitral valve, apical 4 chamber 2D with color-flow Doppler over the tricuspid valve, apical 5 chamber 2D with and without color-flow Doppler over the aortic valve, and subcostal view of the inferior vena cava with sniff. To facilitate reproducibility trainees were taught to obtain views in the pre-specified sequence during each patient exam. Views were selected to identify a predetermined set of six key pathologies, namely left ventricular (LV) dysfunction, pericardial effusion, aortic valve regurgitation, moderate to severe mitral valve regurgitation, moderate to severe tricuspid valve regurgitation, and mitral valve stenosis. A subjective assessment of LV function was based on visual estimation of an ejection fraction (EF): > 50% indicating normal LV function and < 50% indicating LV dysfunction. The LV functional assessment was determined using the parasternal long axis and apical 4 and 5 chamber views. The presence of pericardial effusion was documented regardless of size. Pericardial effusion was assessed using the parasternal long axis and apical 4 and 5 chamber views. Assessment of valvular dysfunction was done utilizing qualitative assessment. With regard to regurgitation, subjective vena contracta width and jet area occupying the respective atria (left atrium for mitral regurgitation and right atrium for tricuspid regurgitation) were utilized. Mitral valve regurgitation was assessed using the parasternal long axis view with color Doppler and the apical 4 chamber view with color Doppler over the mitral valve. Tricuspid regurgitation was assessed using the apical 4 chamber view with color Doppler over the tricuspid valve. Aortic regurgitation was assessed as being present or absent; grading of aortic regurgitation was not performed. The presence of aortic regurgitation was assessed using the parasternal long view with color Doppler and the apical 5 chamber view with color Doppler over the aortic valve. A subjective assessment for mitral stenosis was used, including the presence of subjective thickening and restricted motion of the mitral leaflets and color Doppler demonstrating flow acceleration across the mitral valve. Mitral stenosis was only assessed as present or absent, and grading of severity was not preformed. Assessment of mitral stenosis utilized the parasternal long axis view with and without color Doppler over the mitral valve and apical 4 chamber with and without color Doppler over the mitral valve. Didactic and practical sessions used both English and Kiswahili languages.

### Pre- and post-training assessments

On day 1 immediately prior to initiation of training activities, trainees completed an intake form to gather information about age, sex, professional education, and prior experience performing and interpreting ultrasound images. They were asked to self-assess their knowledge in cardiology and their ability to learn new technical skills as superior, above average, average, or below average. In addition, they took two pre-training knowledge assessments. One knowledge assessment was comprised of ten multiple choice questions focusing on basic cardiac anatomy and physiology (“10Q Anatomy & Physiology” [Additional file 1: Figure S1]). The second knowledge assessment included cardiac ultrasound still images and video clips, with twenty questions focused on identification of major anatomical structures and key pathologies (“20Q Ultrasound Images” [Additional file 2: Figure S2]). On the last day of the in-person training, all trainees underwent a final hands-on observed assessment of their ability to proficiently conduct the scanning protocol and obtain interpretable images, and also independently completed the 10Q Anatomy & Physiology and 20Q Ultrasound Images knowledge assessments.

### Scanning in routine clinical settings

Immediately following the in-person training, Vscan with Dual Probe handheld ultrasound devices (model H544H1AS, GE Healthcare, Chicago, United States) were provided to trainees for use in their usual clinical practice settings. Trainees received guidance to consider conducting a focused cardiac POCUS examination using the pre-specified scanning protocol on patients presenting with signs and/or symptoms of potential cardiovascular etiology. For each patient scanned, the trainee completed a case report form to document the scanning procedure, patient clinical features (limited to age, sex, height, weight, and presenting symptoms/signs) and their interpretation of the images with regard to the key pathologies. For each of the six key pathologies, trainees were asked to indicate whether overall image quality allowed for interpretation of the scan for that pathology; if interpretation was possible, then the trainee was asked to determine whether the pathology was absent or present. For each of the seven scanning views, trainees were instructed to record a representative video. De-identified case report forms were then submitted to the study coordinator and corresponding de-identified images were uploaded to Tricefy, a cloud-based, secure imaging solution. The coordinator then sent blank case report forms and the listing of relevant scans to a designated study cardiologist at JKCI for review. The JKCI study cardiologist, blinded to trainee identity and scan interpretation, reviewed each scan using the Tricefy platform and independently interpreted the key pathologies. In addition, for each of the echocardiographic scanning views, the study cardiologist assessed image quality on a 5-point Likert scale: 0—image not obtained; 1—image quality too poor to permit meaningful interpretation; 3—suboptimal image quality but basic image interpretation possible; 5—good image quality, meaningful image interpretation easy. As a quality control measure, a random selection equal to 25% of scans was interpreted by a separate cardiologist at the Medical University of South Carolina, using the Tricefy platform.

### Analysis

Categorical data were presented as absolute values and proportions. The JKCI study cardiologists’ findings served as the reference standard for interpretation with regard to absence or presence of key pathologies. For key pathologies, agreement, sensitivity, and specificity were calculated for examinations in which both the trainee and the cardiologist had indicated that overall image quality allowed for interpretation of that pathology. Agreement was calculated using Cohen’s kappa statistic (κ), with the following interpretive categories: κ < 0, no agreement; 0.00–0.20, slight agreement; 0.21–0.40, fair agreement; 0.41–0.60, moderate agreement; 0.61–0.80, substantial agreement; 0.81–1.00, almost perfect agreement [23]. Pre- and post-training scores were compared using the Wilcoxon Signed Rank test. The Pearson correlation (continuous variables) or Spearman correlation (ordinal variables) was used to evaluate for associations between trainee baseline characteristics including training test scores (predictors) and outcome variables of image quality (expressed as % of images with quality grade 3 or higher based on cardiologist assessment) or image interpretation (expressed as mean kappa values across the 6 key pathologies).

### Ethics approvals and consent to participate

The IRBs of the Medical University of South Carolina and the Jakaya Kikwete Cardiac Institute reviewed the protocol and determined that this study was exempt from Human Research Subject Regulations under 45 CFR 46.104(d) for educational practices; written informed consent was not applicable for this exempt educational activity.

## Results

### Characteristics of the trainees

Among the eight trainees, six (75%) were male and two (25%) were female (Table 1). The highest level of professional training was Doctor of Medicine (n = 1, 12.5%) followed by Assistant Medical Officer (n = 1, 12.5%); and six (75%) Clinical Officers. None had previously performed a cardiac ultrasound, while two (25%) had previously performed a limited number of antenatal or abdominal ultrasound examinations (Table 1).

**Table 1:** (a) Trainee characteristics, (b) characteristics of patients scanned by trainees

	N (%)
(a)	
Median age in years (range)	39.5 (27–54)
Male	6 (75)
Highest professional education	
Clinical Officer	6 (75)
Assistant Medical Officer	1 (12.5)
Medical Doctor	1 (12.5)
Ever performed an ultrasound	2 (25)^a^
Ever performed a cardiac ultrasound	0 (0)
Ever interpreted a cardiac ultrasound	0 (0)
Self-assessment of ability to learn new technical skills	
Below average	0 (0)
Average	3 (37.5)
Above average	5 (62.5)
Superior	0 (0)
Self-assessment of knowledge base in cardiology	
Below average	0 (0)
Average	7 (87.5)
Above average	1 (12.5)
Superior	0 (0)
(b)	
Age in years (n = 429)	
Median	55 (9–97)
< 20	15 (3.5)
20–39	96 (22.6)
40–59	121 (28.5)
60–79	162 (38.1)
≥ 80	31 (7.3)
Male	138 (32.2)
Symptoms/Signs (n = 429)	
Dyspnea	30 (7.0)
Chest pain	199 (46.4)
Palpitations	140 (32.6)
Lightheaded	40 (9.3)
Cyanosis	2 (0.5)
Hypertension	238 (55.5)
Hypotension	13 (3.0)

^a^1 individual had previously performed approximately 8 antenatal ultrasound examinations and 1 individual had previously performed approximately 10 antenatal plus approximately 10 abdominal ultrasound examinations

### Training period: pre- and immediately post-training written knowledge assessments

On the ten-question multiple choice assessment of basic cardiac anatomy and physiology (10Q Anatomy & Physiology), median number of correct responses pre-training was 7 (range 4 to 9) and post-training was 8.5 (range 8–10, *p* = 0.018). On the twenty-question assessment that incorporated ultrasound images and video clips (20Q Ultrasound Images), median number of correct responses pre-training was 9 (range 5–17) and post-training was 17 (range 10–20, *p* = 0.028).

### Clinical period: characteristics of patients examined by trainees

During a consecutive 9-week period, trainees performed focused cardiac ultrasound examinations on a total of 429 patients at their clinical practice settings. Median number of examinations per trainee was 51 (range 22–97). Characteristics and presenting signs/symptoms of 429 patients are shown in Table 1. Median patient age was 55 (range 9–97). The most common presenting symptoms/signs were hypertension (55.5%), chest pain (46.4%), and palpitations (32.6%).

### Clinical period: quality of cardiac ultrasound images obtained by trainees in their clinical practice

**Fig. 1:**
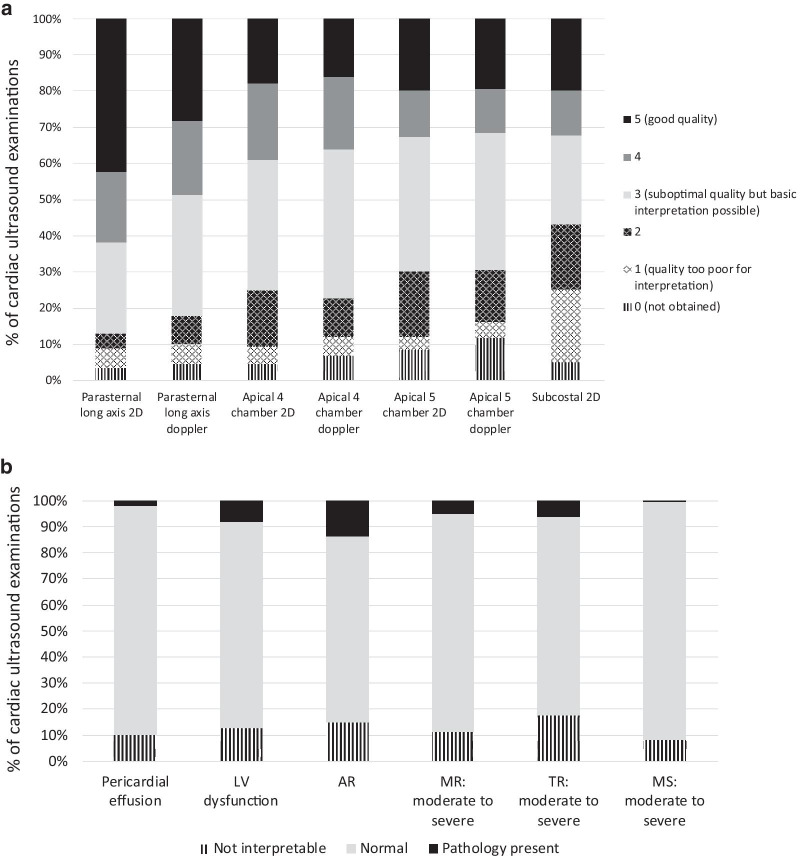
Cardiologist interpretation of image quality for focused cardiac ultrasound examinations performed by trainees. **a** By echocardiographic view; **b** by key pathology

**Fig. 2:**
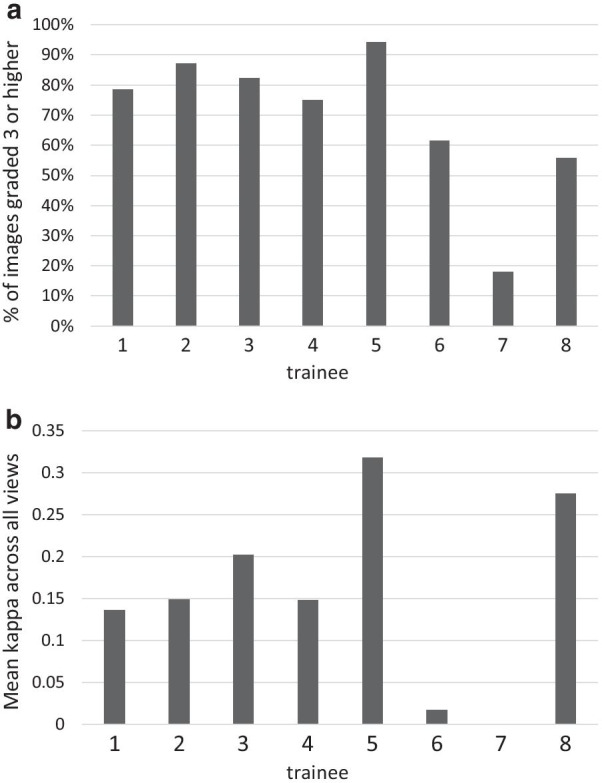
Image quality and interpretation. **a** Image quality: % of all images graded 3 or higher, stratified by trainee; **b** image interpretation: mean kappa values across all views, stratified by trainee

The percentages of images, by scanning view, that were determined by the cardiologists to be of sufficient quality (grade 3 or higher) for interpretation were as follows (Fig. 1a): parasternal long axis 2D 86.9%; parasternal long axis with color Doppler 82.0%; apical 4 chamber 2D 75.0%; apical 4 chamber with color Doppler 77.3%; apical 5 chamber 2D 69.9%; apical 5 chamber with color Doppler 69.4%; subcostal 2D 56.8%. The percentages of scans that were determined by the cardiologists to contain appropriate images of sufficient quality for interpretation as to whether key pathologies were present or absent were as follows: (Fig. 1b): pericardial effusion 89.9%; LV dysfunction 87.3%; aortic regurgitation 85.1%; mitral valve regurgitation 88.9%; tricuspid valve regurgitation 82.4%; presence of mitral valve stenosis 91.8%. By trainee, the overall percentages of images that were determined by cardiologists to be of grade 3 or higher quality ranged from 18.0% (29/161) to 94.2% (356/378), median 76.7%; 5 of 8 trainees achieved 75% or greater interpretable images (Fig. 2a). Score on the post-training 20Q Ultrasound Images assessment (*p* = 0.004) and number of ultrasound examinations performed prior to study training (*p* = 0.039) were each positively associated with image quality in univariate analyses (Table 2).

**Table 2:** Baseline predictors of trainee proficiency in obtaining and interpreting images during the clinical scanning period

Predictor variable, as pertains to trainees	Image quality: % of images graded 3 or higher		Image interpretation: mean kappa across all pathologies	
	Correlation (r)	*p* value	Correlation (r)	*p* value
Trainee age	− 0.398	0.329	− 0.685	0.061
Trainee sex	− 0.050	0.906	0.277	0.507
Highest academic degree	0.655	0.078	0.655	0.078
# of years in clinical practice	− 0.185	0.660	− 0.549	0.158
# ultrasound exams performed prior to training	0.733	0.039	0.452	0.261
Self-described ability to learn new technical skills	0.507	0.200	− 0.056	0.895
Self-described knowledge-base in cardiology	− 0.577	0.134	− 0.577	0.134
Test score: Pre-training 10Q Anatomy and Physiology	0.102	0.810	0.106	0.802
Test score: Post-training 10Q Anatomy and Physiology	0.492	0.215	0.388	0.342
Test score: Pre-training 20Q Ultrasound Images	0.253	0.546	0.217	0.607
Test score: Post-training 20Q Ultrasound Images	0.881	0.004	0.876	0.004

### Clinical period: interpretation of key pathologies based on cardiac ultrasound images obtained by trainees in their clinical practice

Kappa values, sensitivity, and specificity of the trainee interpretation versus the gold standard cardiologist interpretation, by key pathology, are shown in Table 3. Based on cardiologist interpretation, the frequencies of each of the key pathologies among all scans performed were as follows: pericardial effusion 1.9%, LV dysfunction 8.0%, aortic regurgitation 13.4%, mitral valve regurgitation 5.0%, tricuspid valve regurgitation 6.1%, and mitral stenosis 0.2%.

**Table 3:** Agreement, sensitivity, and specificity for trainee interpretations versus gold standard cardiologist interpretation, by key pathology

Key pathology (n assessed)	Kappa (95% CI)	Sensitivity % (95% CI)	Specificity % (95% CI)	Positive predictive value % (95% CI)	Negative predictive value % (95% CI)
Pericardial effusion (n = 367)	− 0.03 (− 0.04, − 0.02)	0 (0, 36.9)	95.8 (93.2, 97.6)	0 (0.00, 19.9)	97.7 (97.7–97.8)
Left ventricular dysfunction (n = 353)	0.17 (0.07, 0.28)	52.9 (36.2, 69.7)	77.1 (72.5, 81.7)	19.8 (11.6–28.0)	93.9 (91.0–96.8)
Aortic regurgitation (n = 343)	0.28 (0.14, 0.41)	29.6 (17.5, 41.8)	93.8 (91.0, 96.6)	47.1 (30.3–63.8)	87.7 (84.0–91.4)
Mitral valve regurgitation: moderate to severe (n = 354)	0.29 (0.14, 0.44)	57.1 (36.0, 78.3)	89.2 (85.9, 92.5)	25.0 (12.7–37.2)	97.1 (95.2–99.0)
Tricuspid valve regurgitation: moderate to severe (n = 334)	0.42 (0.25, 0.59)	59.1 (38.6, 79.6)	93.3 (90.5, 96.1)	38.2 (21.9–54.56)	97.0 (95.1–98.9)
Mitral valve stenosis: moderate to severe (n = 371)	0 (0, 0)	N/A	86.5 (82.6, 89.8)	N/A	N/A

Agreement between trainee and cardiologist was moderate for interpretation of tricuspid valve regurgitation; fair for aortic regurgitation and mitral valve regurgitation; slight for LV dysfunction; and poor for the presence of mitral stenosis and pericardial effusion. Additional file 3: Figure S3 through Additional file 5: Figure S5 are examples of trainee-obtained images that were of sufficient quality to be interpretable, yet the trainee did not detect a pathology that was present based on the cardiologist’s interpretation. Scans from eight patients showed a pericardial effusion, but none were detected by trainees. Among 34 patients with LV dysfunction, 18 (52.9%) were interpreted as such by trainees, and among 319 patients with normal LV function, 73 (22.9%) were incorrectly interpreted by trainees as having LV dysfunction. By trainee, mean kappa values across the 6 key pathologies ranged from 0 (no agreement) to 0.32 (fair agreement) (Fig. 2b). There was a significant association between score on the post-training 20Q Ultrasound Images assessment and mean kappa value across the 6 key pathologies in univariate analysis (*p* = 0.004).

There was almost perfect agreement for image interpretation between the JKCI cardiologist and MUSC cardiologist, with kappa 0.84 (95% CI 0.75–0.92).

## Discussion

During this pilot program, eight clinicians participated in a 5-day training in the performance and interpretation of cardiac POCUS examinations, and then conducted a total of 429 independent examinations in their usual clinical care settings. Key training period findings were the broad range in pre-training knowledge and the significant increases in cardiac ultrasound-specific knowledge based on post- versus pre-training scores on the 20Q Ultrasound Images assessment. During the clinical scanning period the majority of trainees were able to obtain imaging proficiency, such that 75% or more of obtained images allowed meaningful interpretation. These results are generally comparable to a study demonstrating that third-year medical students in Canada could obtain an overall accuracy of 82% in obtaining 8 prespecified images after a relatively brief training program [20].

However, in our study, agreement between trainee and cardiologist interpretation of key pathologies was relatively poor, not only for detection of valvular pathologies, considered an advanced skill, but also for detection of LV dysfunction and pericardial effusion which are typically more straightforward to recognize. In this respect our findings differ from some previously published experiences. In the aforementied study conducted in Canada by Cawthorn et al., the mean echo interpretive score was approximately 80% [20]. The differences in interpretive ability between our trainee cohort and that described by Cawthorn may be related to differences within the cardiac POCUS training programs or differences in fundamental knowledge surrounding cardiac anatomy and pathology. Within our training program there were 2 didactic sessions totaling approximately 5 h over 2 days. However, within the Cawthorn et al. study, students received 4 didatic sessions each for 2 h over a 2 week period or 3 electronic modules totaling 10 h that could be accessed as much as the study participants desired. Both the reduction of total didactic time as well as the condensing of that time into a shorter window may have led to the reduced accuracy in image interpretation among our trainee cohort. Additionally, while our study did dedicate more time to “hands on” image acquisition skills (approximately 20 h vs 4 h), this was largely spent in one large group as opposed to multiple smaller groups. Thus while more total time was spent, the larger group number may have resulted in less “hands on” time per trainee. Some studies of non-physician use of cardiac POCUS in African settings have focused only on identification of marked valvular abnormalities associated with rheumatic heart disease, a narrower scope of investigation than addressed in our study [24, 25]. Our findings are in some respects reminiscent of those by Sanyahumbi and colleagues who found that, in instances of “missed” rheumatic heart disease, the images acquired were adequate to make the diagnosis but were not interpreted correctly by the Clinical Officer [24]. In our study, the Clinical Officers read images on the small Vscan screen at point of care (per written study procedures), whereas the cardiologists viewed images via Tricefy on a larger desktop computer screen, at their convenience—the extent to which these differences contributed to differences in image interpretation by trainees and cardiologists is unclear.

Another limitation of our study is the relatively low prevalence of each of the key pathologies during the clinical period. From an analysis perspective this resulted in broad confidence intervals around sensitivity point estimates, and it also could have impeded trainee learning and self-improvement during the clinical period because of lack of exposure to a spectrum of abnormal and normal images. On the other hand, the clinical period reflected “real life” in the trainees’ usual clinical care environments, and therefore the results may be generalizable to other similar settings, underscoring the challenges in maintaining and building proficiency in image interpretation.

Results during the clinical scanning period revealed proficiency differences between trainees in obtaining and interpreting images. Score on the immediate post-training assessment that incorporated cardiac ultrasound images (20Q Ultrasound Images) was strongly associated with subsequent proficiency in obtaining quality images and with accurate image interpretation during the clinical scanning period. This type of assessment instrument may help to identify individuals who would benefit from additional training. Within our cohort, higher assessment scores and higher proficiency in image aquisition appeared to correlate with higher levels of formal training and education.

Thus, while a key limitation of this pilot study was its small sample size, our findings suggest that a short training program (e.g. 5 days) may be sufficient to train most clinicians at the Clinical Officer level or above to proficiently obtain, but not to accurately interpret, basic cardiac ultrasound images. Proficiency in cardiac ultrasound image interpretation may require a greater level of existing knowledge prior to a short ultrasound training program, or a more intensive ultrasound training program. Advances in wireless image transfer might allow for hybrid clinical approaches that combine point-of-care imaging by a larger cadre of clinicians with real-time or near-real-time interpretation by a specialist not physically co-located with those clinicians.

## Supplementary Information

**Additional file 1. Figure S1:** 10Q Anatomy & Physiology assessment.

**Additional file 2. Figure S2:** 20Q Ultrasound Images assessment.

**Additional file 3. Figure S3:** Example image: image quality sufficient for detection of tricuspid regurgitation, but abnormality not detected by trainee.

**Additional file 4. Figure S4:** Example image: image quality sufficient for detection of aortic regurgitation, but abnormality not detected by trainee.

**Additional file 5. Figure S5:** Example image: image quality sufficient for detection of aortic regurgitation, but abnormality not detected by trainee.

## Data Availability

The images generated and analysed during this study are not publicly available for reasons of patient confidentiality, but de-identified images are available from the corresponding author on reasonable request and with an appropriate data sharing agreement.
