# Supplementary material for: Feasibility of point-of-care cardiac ultrasound performed by clinicians at health centers in Tanzania
Source: BMC Cardiovasc Disord. 2021 May 12;21:239. doi: 10.1186/s12872-021-02045-y (PMC8117304; doi:10.1186/s12872-021-02045-y)
Supplement: Supplementary file 1 — Additional file 1. Figure S1: 10Q Anatomy & Physiology assessment. [file 12872_2021_2045_MOESM1_ESM.pdf]

Trainee ID: **TR - __ __**

Date of CRF Completion: __ __ /__ __ __/ __ __ __ __

VScan: Pre-Training Knowledge Assessment: Multiple Choice

**Pre-Training Knowledge Assessment: Multiple Choice**<br>The following assessment is multiple choice.<br>For each question, please select **one** best response.<br>Please indicate your selection with an "x."	
1 The normal human heart has how many chambers?	□ One<br>□ Two<br>□ Three<br>□ Four
2. In the normal human heart, which cardiac chamber is largest and has the thickest walls?	□ Right ventricle<br>□ Left ventricle<br>□ Right atrium<br>□ Left atrium
3. The left atrium receives blood from which of the following structures?	□ Aorta<br>□ Pulmonary veins<br>□ Right atrium<br>□ Left atrium
4. What is the name of the valve that separates the left atrium from the left ventricle?	□ Aortic valve<br>□ Pulmonic valve<br>□ Mitral valve<br>□ Tricuspid valve
5. The left ventricle pumps blood into what structure?	□ Aorta<br>□ Pulmonary Veins<br>□ Right atrium<br>□ Right ventricle
6. The right atrium receives blood from which of the following structures?	□ Aorta<br>□ Pulmonary veins<br>□ Right atrium<br>□ Superior and inferior vena cava
7. What is the name of the valve that separates the right atrium from the right ventricle?	□ Aortic valve<br>□ Pulmonic Valve<br>□ Mitral Valve<br>□ Tricuspid Valve
8. What is the normal wall thickness for the inter-ventricular septum and posterior LV wall?	□ 2 to 5 mm<br>□ 6 to 12 mm<br>□ 14 to 17 mm
9. When associated with pericardial effusion, collapse of the right atrium and right ventricle during diastole can be an indication of:	□ Left ventricular hypertrophy<br>□ Cardiac tamponade<br>□ Severe aortic valve stenosis<br>□ Severe mitral valve stenosis
10. What is the normal left ventricular ejection fraction?	□ 10 to 20%<br>□ 30 to 44%<br>□ ≥ 55%
