# Supplementary material for: Feasibility of point-of-care cardiac ultrasound performed by clinicians at health centers in Tanzania
Source: BMC Cardiovasc Disord. 2021 May 12;21:239. doi: 10.1186/s12872-021-02045-y (PMC8117304; doi:10.1186/s12872-021-02045-y)
Supplement: Supplementary file 2 — Additional file 2. Figure S2: 20Q Ultrasound Images assessment. [file 12872_2021_2045_MOESM2_ESM.pdf]

Trainee ID: TR - __ __

Date of CRF Completion: __ __ /__ __ __ / __ __ __ __

VScan: Pre-Training Knowledge Assessment: Vignettes

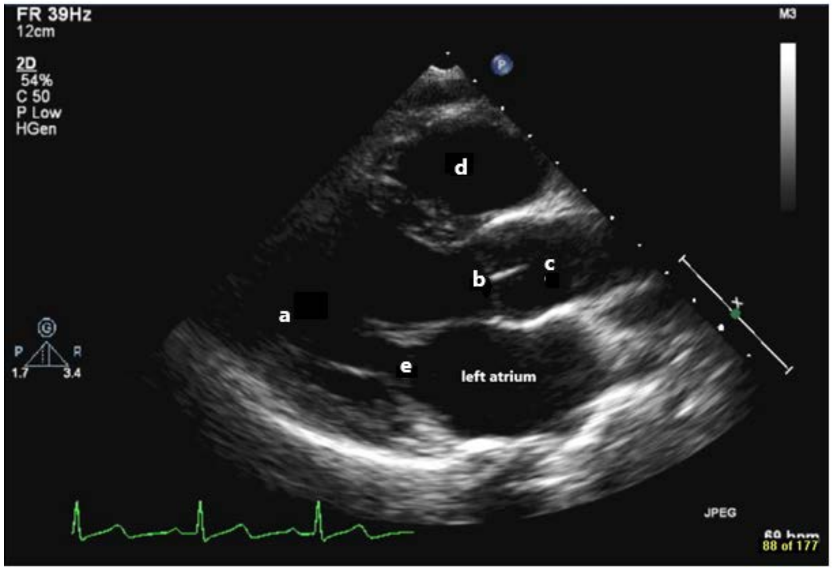

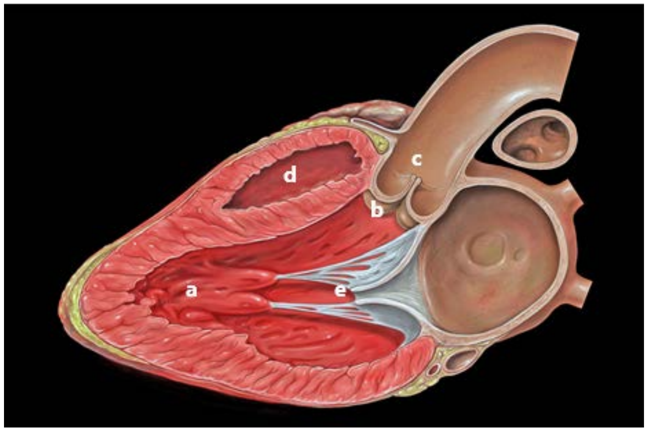

1.) Please identify the structures of the heart labelled in the parasternal long axis view above (left). Please feel free to refer to the drawing of the heart on the right if it is helpful for orientating yourself to the cardiac ultrasound image on the left.

a.) ____________________________

b.) ____________________________

c.) ____________________________

d.) ____________________________

e.) ____________________________

VScan: Pre-Training Knowledge Assessment: Vignettes

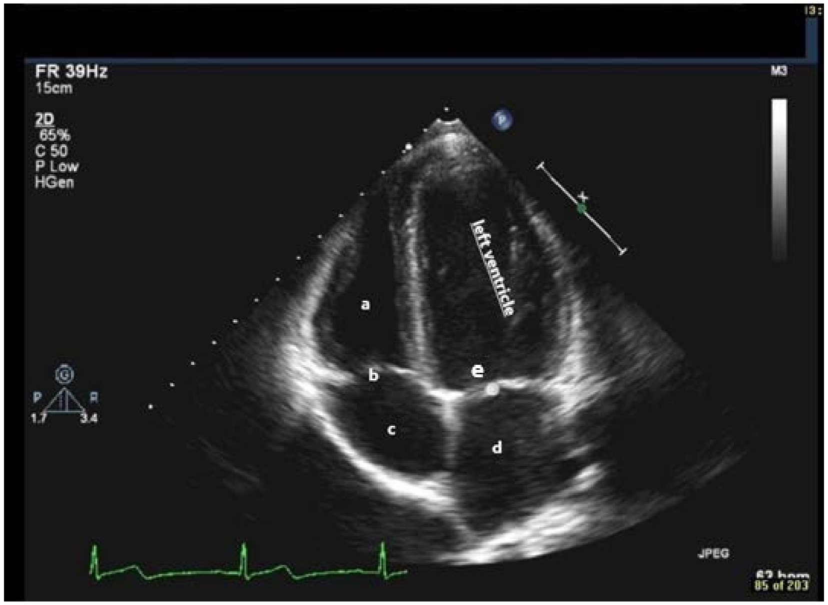

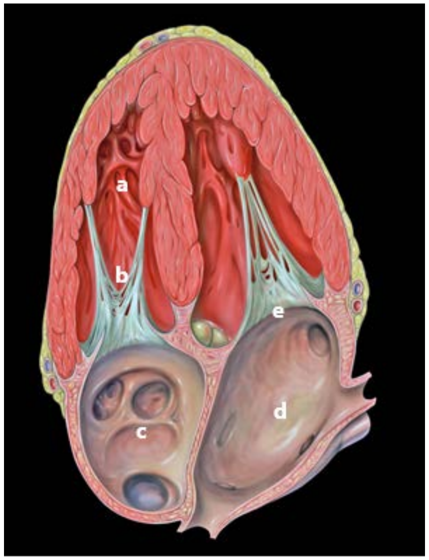

2.) Please identify the structures of the heart labelled in the apical 4 chamber view above (left). Please feel free to refer to the drawing of the heart on the right if it is helpful for orientating yourself to the cardiac ultrasound image on the left.

a.) ____________________________

b.) ____________________________

c.) ____________________________

d.) ____________________________

e.) ____________________________

Trainee ID: TR - __ __

Date of CRF Completion: __ __ /__ __ __ / __ __ __ __

VScan: Pre-Training Knowledge Assessment: Vignettes

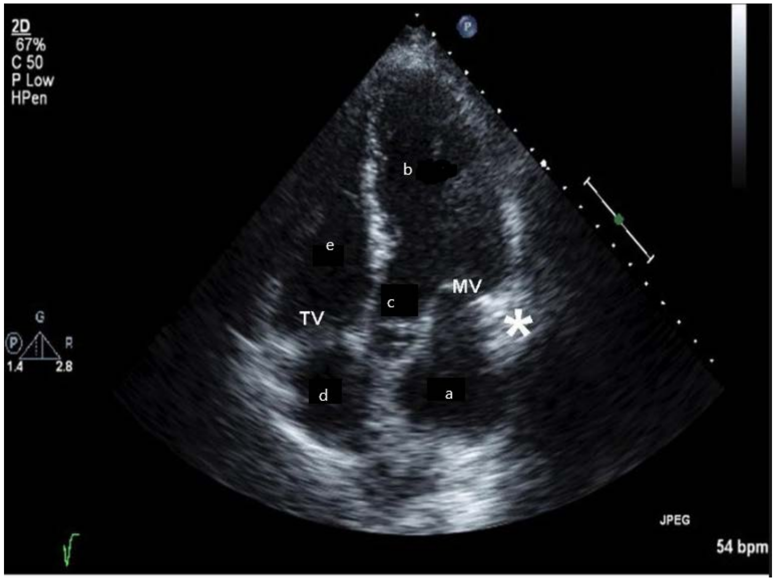

3.) Please identify the structures of the heart labelled in the apical 5 chamber view above.

a.) ______________________________

b.) ______________________________

c.) ______________________________

d.) ______________________________

e.) ______________________________

VScan: Pre-Training Knowledge Assessment: Vignettes

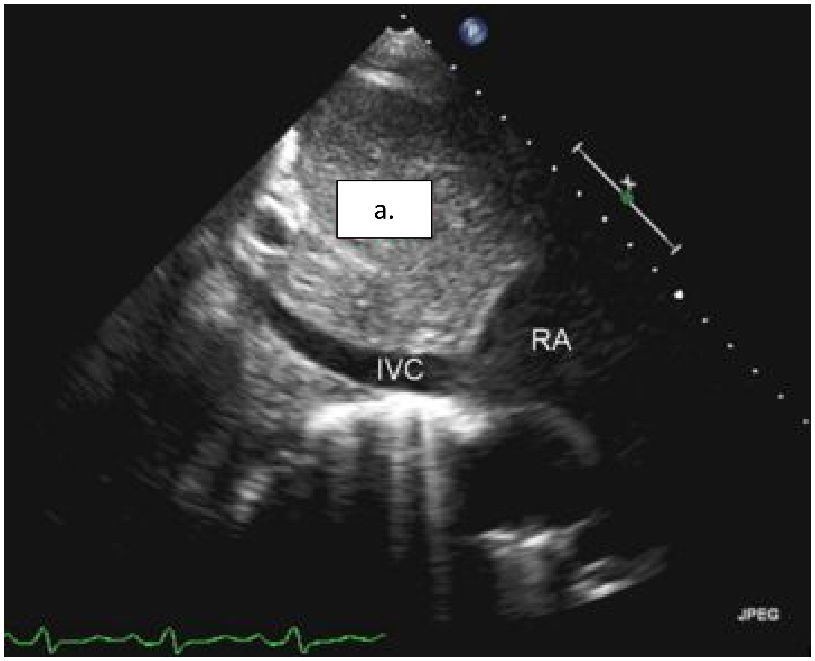

4.) Please label this anatomical landmark of importance in the subcostal (inferior vena cava) view.

a.) ____________________________

VScan: Pre-Training Knowledge Assessment: Vignettes

Please refer to the videos on screen for the following 4 questions.

5.) Please evaluate the left ventricular function in the images on screen. In images a and b, is left ventricular function:

a.) Normal

b.) Mildly suppressed

c.) Severely suppressed

6.) Please evaluate the left ventricular function in the images on screen. In images c and d, is left ventricular function:

a.) Normal

b.) Mildly suppressed

c.) Severely suppressed

7.) In images e and f please assess the presence or absence of mitral regurgitation.

a.) None – Mild Mitral Regurgitation

b.) Moderate-Severe Mitral Regurgitation

8.) In images g and h, is the mitral valve stenosis present or absent?

a) Mitral stenosis present

b.) Mitral stenosis absent
