# Supplementary figures and images for: Feasibility of point-of-care cardiac ultrasound performed by clinicians at health centers in Tanzania
Source: BMC Cardiovasc Disord. 2021 May 12;21:239. doi: 10.1186/s12872-021-02045-y (PMC8117304; doi:10.1186/s12872-021-02045-y)

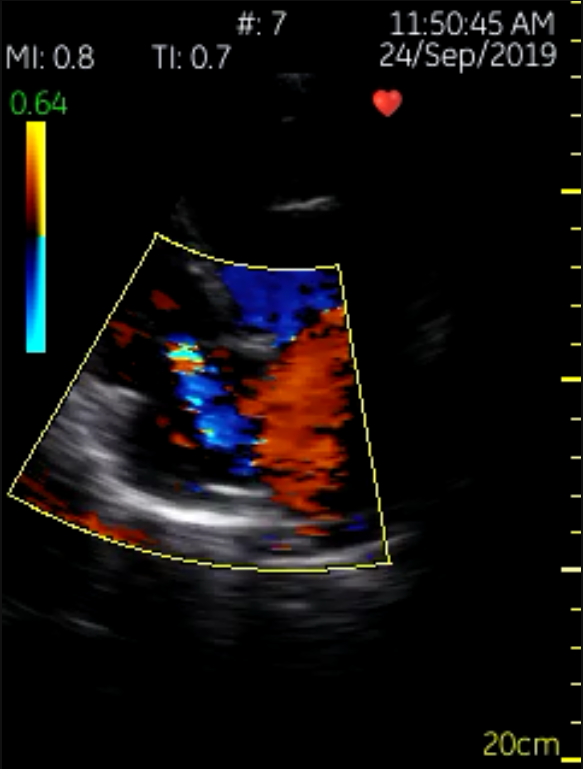

Supplement: Supplementary file 3 — Additional file 3. Figure S3: Example image: image quality sufficient for detection of tricuspid regurgitation, but abnormality not detected by trainee. [file 12872_2021_2045_MOESM3_ESM.png]

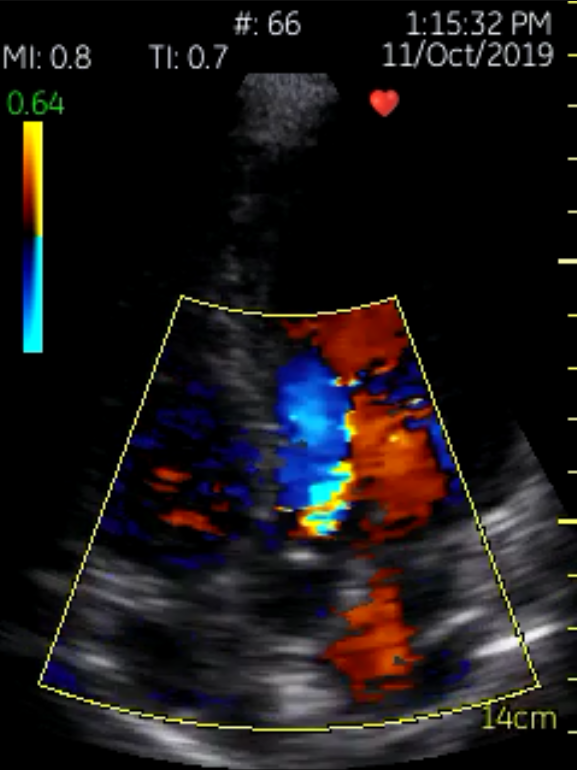

Supplement: Supplementary file 4 — Additional file 4. Figure S4: Example image: image quality sufficient for detection of aortic regurgitation, but abnormality not detected by trainee. [file 12872_2021_2045_MOESM4_ESM.png]

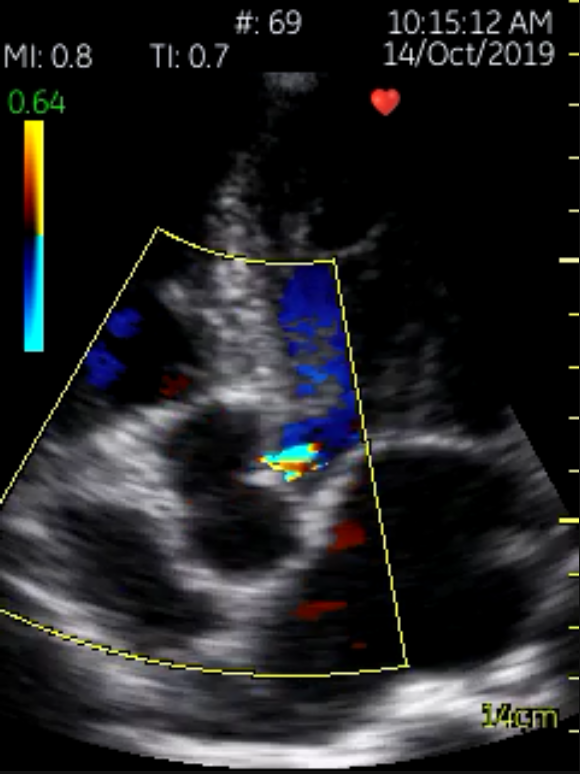

Supplement: Supplementary file 5 — Additional file 5. Figure S5: Example image: image quality sufficient for detection of aortic regurgitation, but abnormality not detected by trainee. [file 12872_2021_2045_MOESM5_ESM.png]
